# Case report: Endovascular treatment of two scalp arteriovenous malformation cases *via* direct percutaneous catheterization: A case series

**DOI:** 10.3389/fneur.2022.945961

**Published:** 2022-07-25

**Authors:** Yuan Shi, Peixi Liu, Yingtao Liu, Kai Quan, Peiliang Li, Zongze Li, Wei Zhu, Yanlong Tian

**Affiliations:** ^1^Department of Neurosurgery, Huashan Hospital, Fudan University, Shanghai, China; ^2^Institute of Neurosurgery, Fudan University, Shanghai, China; ^3^National Center for Neurological Disorders, Shanghai, China; ^4^Shanghai Clinical Medical Center of Neurosurgery, Shanghai, China; ^5^Shanghai Key Laboratory of Brain Function and Restoration and Neural Regeneration, Shanghai, China; ^6^Department of Radiology, Huashan Hospital, Fudan University, Shanghai, China

**Keywords:** scalp arteriovenous malformation, scalp arteriovenous fistula, endovascular embolization, direct percutaneous puncture, case report

## Abstract

**Background:**

Scalp arteriovenous malformations (AVM) are rare vascular malformations reported only in small case series. Scalp AVMs usually present with symptoms, including headache, tinnitus, epilepsy, cerebral ischemia, and necrosis of the scalp, which can cause functional, cosmetic, and psychological problems. There are many difficulties in the treatment of scalp AVM because of its complex characteristics of vascular anatomy, non-uniform structure, and intracranial-extracranial anastomosis.

**Case description:**

To illustrate the endovascular treatment of scalp AVM *via* direct percutaneous puncture while traditional arterial and venous approaches were not available. In this report, access was obtained through a direct puncture of the enlarged frontal vein. Onyx-18 was injected through a microcatheter to occlude draining veins, fistulous connection, and the feeders. An 18-gauge indwelling needle was inserted into draining veins directly. Postembolization angiography demonstrated complete sAVM occlusion immediately and no non-targeted embolization. At a 1-year follow-up, no procedure-related complications and evidence of recurrence were observed.

**Conclusion:**

The technique of endovascular embolization *via* direct percutaneous puncture approach is safe, rapid, and effective for specific sAVM. Treatment options should be made in terms of size, vascular anatomical characteristics of the lesions, patient's preference, cosmetic factors, and available expertise.

## Introduction

Scalp arteriovenous malformations (sAVM), also known as cirsoid aneurysms or scalp arteriovenous fistulas (AFV), are anomalous connections between superficial arteries and veins without capillaries. The pathological mechanism of scalp AVMs is still unclear. These cases are relatively rare and most pediatric cases are congenital.

Many factors, such as trauma, craniotomy, hair implantation, infection, and inflammation, are involved with the etiology of scalp AVMs (1). The AVMs have been reported to account for 8.1% of all AVMs (2). These AVMs are pulsatile lesions with headache, tinnitus, vascular murmur, local bleeding, and necrosis of the scalp (3). In addition, epilepsy and cerebral ischemia could be caused by abnormal shunting of common carotid blood flow (4). The clinical symptoms could worsen while the lesions are enlarged. The mass developed from vascular abnormalities can cause functional, cosmetic, and psychological problems.

In this article, we reported a case series of scalp AVMs treated with Onyx *via* a direct percutaneous puncture approach.

## Case description

This study was approved by the Institutional Review Board of Ethics of our hospital. Both patients agreed to be photographed and understood that their identity could be revealed on camera and in the content of the publication. CARE Checklist was implemented in this case report.

### Case 1

#### History and examination

The first case is a 16-year-old male patient with complaints of progressive enlargement of forehead mass for 10 years. The lesion presented as a soft, pulsatile mass, and the border was not clear. Bowed head and physical exercises could increase the volume of mass. Other neurological symptoms are negative. The patient had no family history of vascular malformations, no history of head trauma, and febrile illness. Local hospital magnetic resonance imaging (MRI) demonstrated a subcutaneous flow void sign in the frontal region, which indicated abnormal vessel mass in the lesion.

#### Endovascular treatment

**Figure 1:**
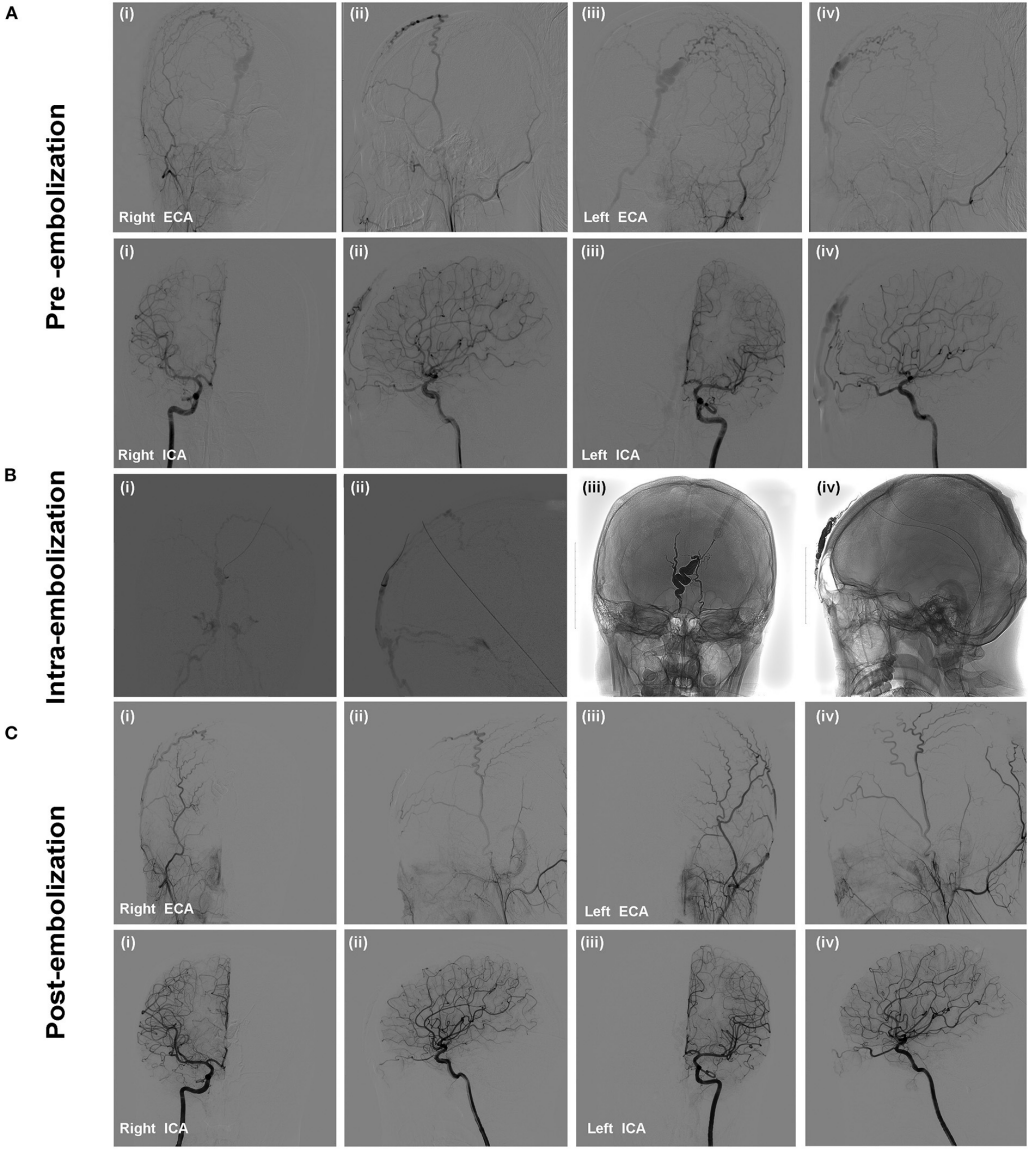
The patient presented with a pulsatile forehead mass without specific medical history. Selective cerebral DSA confirmed the presence of scalp AVM. Angiographic images showed that the feeding arteries of the lesion were bilateral frontal branches of STAs and ophthalmic arteries **(A)**. Onyx-18 liquid embolic material was injected to embolize the draining veins, fistulous connection, and feeding arteries. **(B)** Angiography was performed *via* microcatheter (i,ii); Penetration and solidification of Onyx after embolization (iii,iv); **(C)** Post-embolization angiography demonstrated the scalp AVM was occluded completely without non-targeted vessels embolization.

Cerebral diagnostic digital subtraction angiography (DSA)was performed under local anesthesia after the patient was admitted to our department. DSA confirmed the presence of frontal sAVM with feeding arteries from bilateral frontal branches of STAs and ophthalmic arteries and draining into orbital veins and facial veins (Figure 1A). We analyzed sAVM angio-architecture and a decision was made for treatment *via* endovascular embolization.

**Figure 2:**
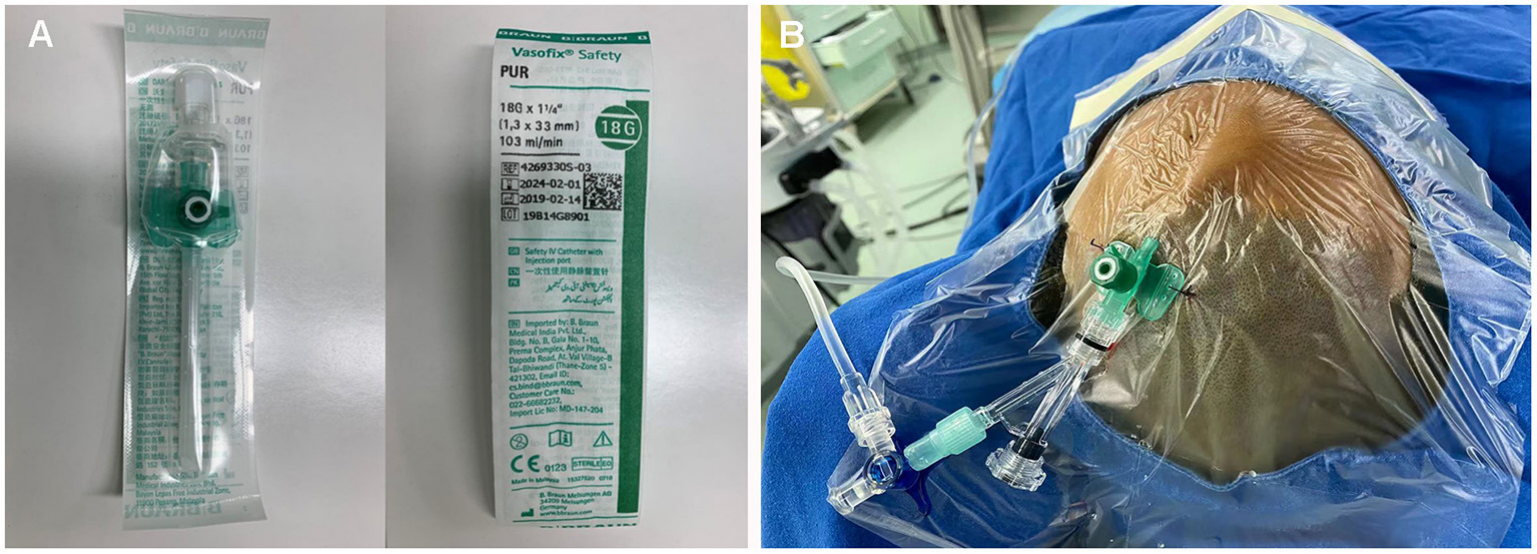
**(A,B)** Direct percutaneous catheterization of the draining veins was performed with an 18-gauge needle.

The embolization was performed under general anesthesia. A 5-Fr H1 catheter (Cook Medical, Bloomington, U.S.A) was targeted at the right external carotid artery (ECA) as a monitoring catheter through the sheath. In consideration of the location of the sAVM, access was obtained through direct puncture of the enlarged frontal vein with an 18-gauge needle and sutured in place (Figures 2A,B). Next, a microcatheter (Echelon 10; Covidien, Irvine, California, USA) was navigated through the 18-G venous indwelling needle. The contrast was injected to confirm the position of the microcatheter tip within the draining vein and was close to the fistulous connection (Figures 1Bi,ii). The microcatheter was flushed with dimethyl sulfoxide(DMSO) (0.3 ml). Onyx-18 (ev3 Endovascular; Medtronic, Minneapolis, Minnesota, USA) liquid embolic material (2.5 ml) was then carefully injected through the microcatheter under a road map and allowed to diffuse to fill the draining veins, fistulous connection, and the feeders successfully (Figures 1Biii,iv). Post-embolization angiography of bilateral internal carotid arteries (ICA) and ECAs demonstrated complete sAVM occlusion immediately after Onyx-18 injection (Figure 1C).

#### Postoperative course

**Figure 3:**
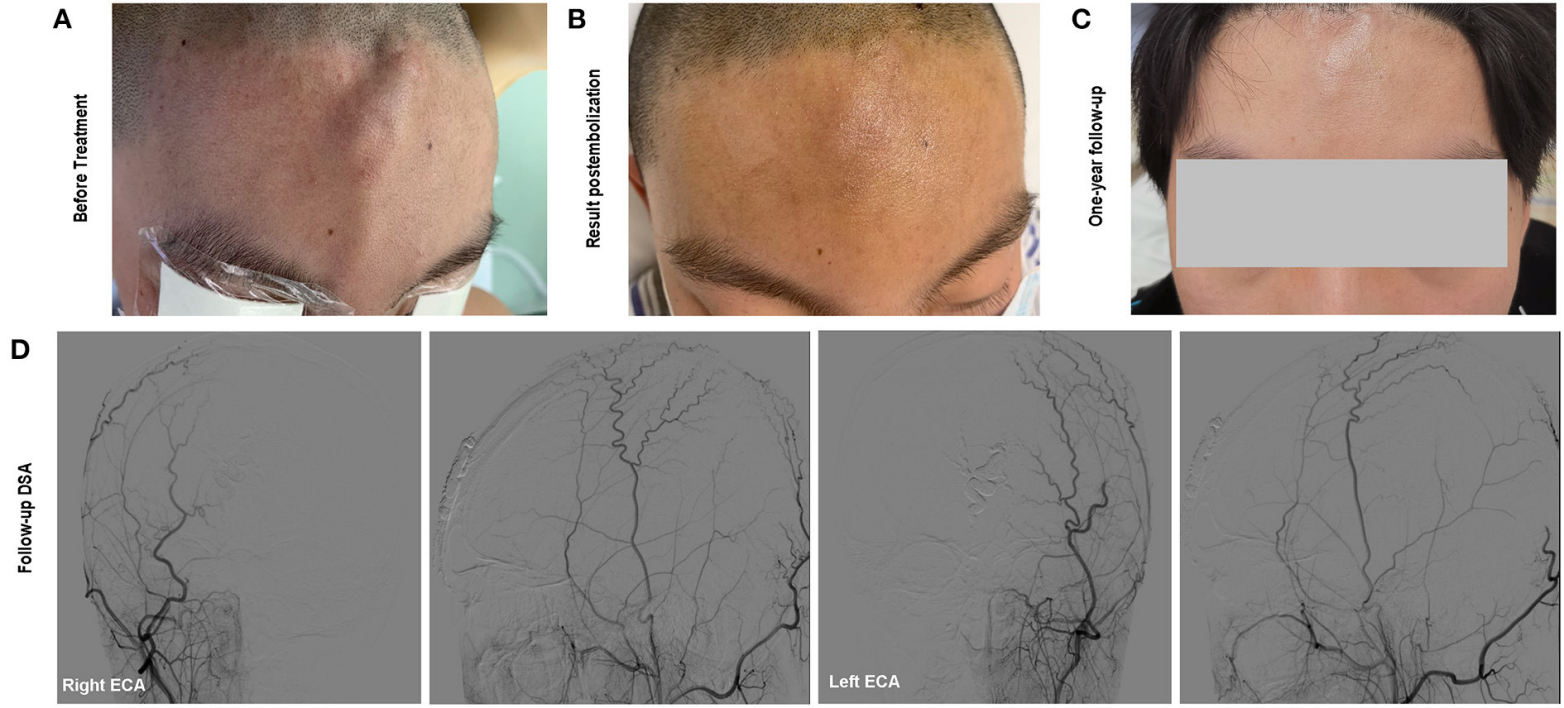
**(A)** The soft and pulsatile lesion with no clear border before treatment; **(B)** Immediate status of the lesion postembolization; **(C)** One-year follow-up showed no evidence of recurrence and scalp necrosis. Onyx was not visible beneath the skin; **(D)** Follow-up angiography showed complete occlusion of the scalp AVM.

The size of the mass decreased immediately after the embolization and did not present any more pulsation (Figures 3A,B). The lesion was pressurized bondage slightly after embolization for 12 h. One-year follow-up cerebral angiography demonstrated no evidence of the sAVM recurrence (Figure 3D). No skin necrosis was observed (Figure 3C).

### Case 2

#### History and examination

A 23-year-old female patient presented with a 5-year-history of right occipital tender, pulsatile mass, and continued to enlarge recently. Neurological examination showed no pathological signs. The patient had no history of head trauma, hair transplantation, and other pertinent histories. MRI showed an abnormal vascular mass of the lesion.

#### Endovascular treatment

**Figure 4:**
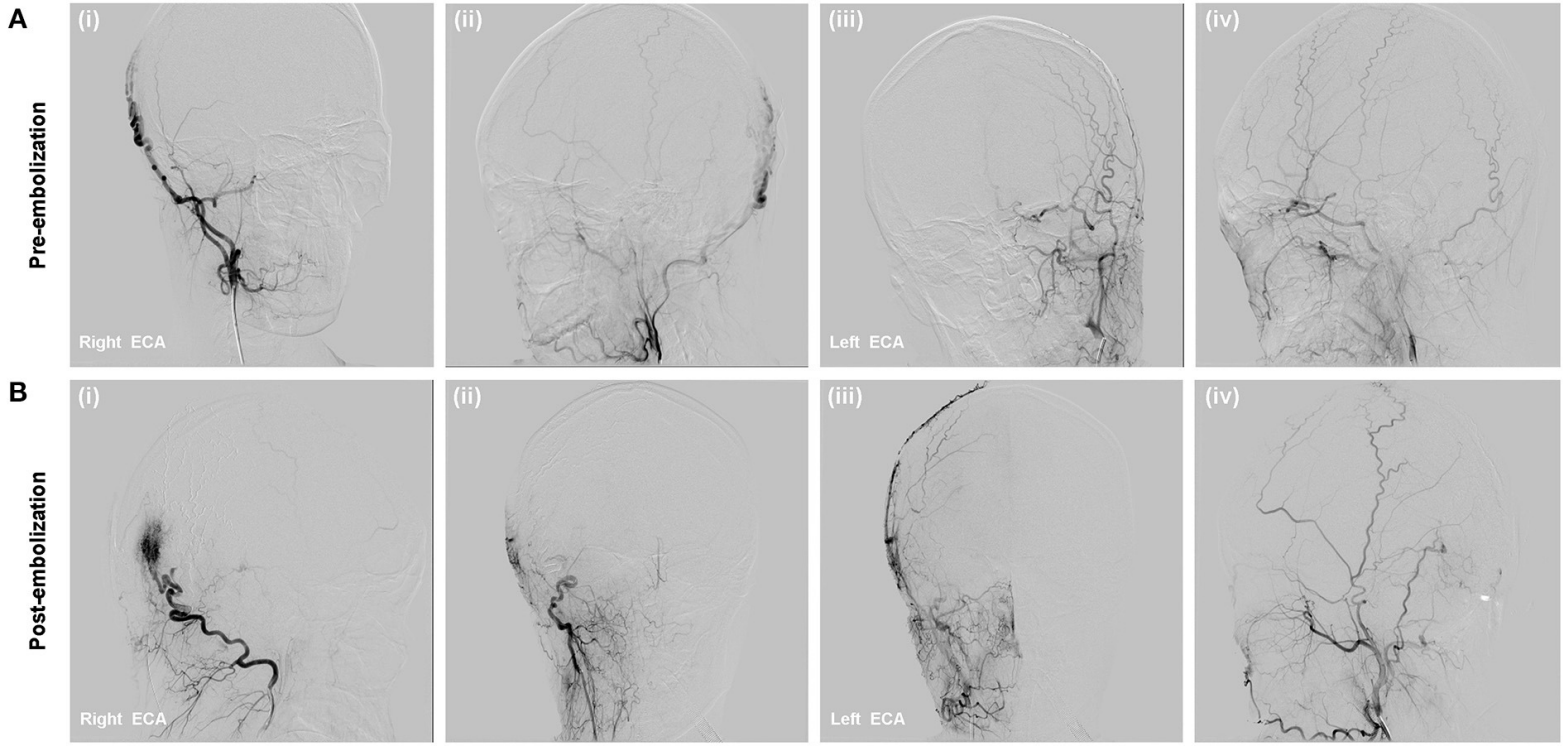
**(A)** DSA showed right occipital high flow scalp AVM fed from the right OA branches and drained to the external jugular vein; **(B)** Onyx-18 was injected *via* direct percutaneous puncture approach (i,ii); Immediate complete occlusion of the scalp AVM post-embolization (iii,iv).

In our department, super-selective diagnostic DSA was performed. DSA confirmed the diagnosis of high-flow right occipital sAVM and revealed arterial supply from right OA, whereas the temporal and occipital tributaries of the external jugular vein were acting as drainers (Figure 4A). Then, we planned to perform treatment through a venous approach.

After cannulation into the right femoral vein was achieved using a 6-Fr guiding sheath, the 6-Fr Envoy guiding catheter (Codman, Miami Lakes, Florida, USA) was positioned within the right external jugular vein (EJV). Two microcatheter (Echelon 10) were advanced and placed in the distal primary targeted vessel assisted with microwire. Angiographic images demonstrated that the microcatheter tip was far from the malformation mass, which could lead to poor Onyx diffusion and microcatheter trapping. Thus, direct percutaneous catheterization under the guidance of the roadmap was performed after the occipital region was sterilized. Contrast injection confirmed an 18-gauge needle was placed in the draining vein. The 18-gauge needle system was flushed with DMSO, and Onyx-18 liquid embolic material (13 ml) was injected to completely occlude the nidus, partial feeding arteries, and draining veins, which was confirmed by post-embolization angiography. Post-embolization angiography showed that the fistula was occluded and no non-targeted embolization (Figure 4B), with preservation of the main intracranial branches of bilateral ICAs.

#### Postoperative course

The occipital lesion was wrapped with pressure dressing after the removal of the needle. At the 1-year follow-up visit, complete resolution of the pulsatile mass with no cutaneous necrosis was found.

## Discussion

Scalp arteriovenous malformations (sAVM) is an abnormal fistulous connection between arteries and veins without an intervening capillary bed in the subcutaneous layer of the scalp (5). They used to be called aneurysm cirsoide, aneurysma serpentinum, plexiform angioma, and scalp AVF (6). The lesions are rare and little is known about their etiology and pathology. Most sAVM is spontaneous, however, it can be acquired after hair transplantation, head trauma, or craniotomy (1). sAVM often presents as progressively enlarged pulsatile mass (with 94.4% of patients) associated with headaches (25.3%), tinnitus (20.2%), bruits, local pain, epilepsy, hemorrhage, and scalp necrosis (7).

The sAVM is often detected by physical examination and the diagnosis is proven by computed tomography angiography (CTA) and magnetic resonance angiography (MRA) (8). Most sAVMs are located in frontal, temporal, and occipital regions, and their feeding arteries often arise from the superficial temporal artery (STA) or occipital artery (OA), and then, drain into the extracranial venous system. Statistics results indicate that there are more than one distinct feeding arteries in 54.2% of cases (7). The gold diagnostic standard is selective DSA. Catheter angiography is recommended to differentiate sAVM from other diseases, including venous malformation, hematoma, lipoma, cyst, abscess, lymphadenopathy, and tumor with abundant blood supply (5, 9, 10).

The sAVM is a kind of intractable vascular disease because of its specific characteristics, including heterogeneous vascular anatomy, high flow shunt, intracranial-extracranial anastomosis, and, probably, cosmetic demands. DSA can reveal whether there is a co-occurrence of intracranial vascular abnormalities and intracranial communication of sAVM, which affect treatment strategies. Besides, preoperative angiographic evaluation can be used for the assessment of feeders, draining pattern, amount of fistular connections, and abnormal shunt blood volume, which contribute to avoiding complications (11). Thus, DSA evaluation is essential before surgical and endovascular treatment.

Surgery used to be the first choice in the past years (12). However, endovascular embolization and surgery combined with embolization have been used more frequently with the development of endovascular technologies and a new type of liquid embolic agent (13). Some other treatments have also been reported, like simple fistular ligation and sclerotherapy with ethanol, which is suitable for small lesions with low blood volume and bleeding probability (14).

A previous literature review indicated that open surgery is preferred for large cases (>4 cm) with multiple feeding arteries (15, 16). The key steps of surgical resection include the design of flap incision, feeders controlling, and *en bloc* excision without scalp necrosis and massive blood loss (17). Possible complications are hematoma, seroma, alopecia, and flap necrosis. Surgical excision is limited by the risk of normal scalp vascularization impairment and severe blood loss, even though a better cosmetic result may be obtained (18).

Surgical resection of sAVM used to be the most common and successful method before the endovascular era. Recently, endovascular therapy has become the most extensively accepted as a single treatment or combined with open surgery for some complex cases. Endovascular embolization is preferred for small lesions (<4 cm) and fewer feeding/draining vessels (19, 20). It has also been used to reduce excessive hemorrhage during surgical procedure. Previous work by Kawamata et al. showed that embolization of both feeders and nidus before surgery is more effective than embolization of the feeders alone to control the hemorrhage during excision (21). Coils and Onyx are most frequently used in the treatment of sAVM. The risks of endovascular therapy include scalp ulceration, non-targeted vessel embolization, systemic embolization, and local tattooing (22, 23). To minimize the above-mentioned risks, the method of “armored concrete” embolization (coils and Onyx are used) (24) and the balloon-protect technique *via* transvenous approach have been reported (25).

The most frequently used approach to embolization is femoral trans-arterial and transvenous routes. However, direct puncture of the fistular is recommended as an alternative option, while feeding/draining vessels are tortuous (26, 27). In the direct puncture technique, the venous pouch is targeted. During the embolization, external compression is performed, which causes the reflux of the Onyx into the nidus (28). Our experience showed direct puncture could reduce cost and operative time compared with the trans-arterial approach, especially for small and low-flow lesions.

In this paper, we reported two cases of sAVM embolization by direct percutaneous catheterization of the draining veins. A “plug” is usually made to prevent the regurgitation of embolic agents during the embolization of intracranial vascular malformations (brain AVM or brain AVF). In our cases, Onyx was injected under temporary compression of draining vein by fingers, which retrogradely allowed penetration of the agent into the feeding arteries, nidus, and venous drainage. Due to the short access distance, there was little difficulty in extubation, and the risk of bleeding during extubation was low. The two cases were superficial scalp AVMs with moderate blood flow. The onyx could be injected into nidus effectively. Besides, we did not make the plug-in consideration of cosmetic and economic factors. For cases with high blood flow, the double-lumen balloon catheter and cooker technology could be used (29). The interventional access of the patients through an artery or vein was not possible, which did not facilitate adhesion and solidification of Onyx or penetration of Onyx into the shunt. In our series, no procedure-related complications were recorded. At a 1-year follow-up, none of the symptoms had recurred.

## Limitations

There are some limitations to this study. Scalp AVMs are rare lesions and this was a retrospective single-center study with a small number of patients. However, our experience demonstrated that endovascular treatment *via* direct puncture was safe and effective while traditional approaches were not available.

## Conclusion

In our series of cases, we reported technical details of scalp AVM endovascular therapy *via* a direct percutaneous puncture approach, which indicates that scalp AVM embolization with Onyx is safe and effective. There is still no consensus on the treatment of the scalp AVM. Treatment options should be made in terms of size, vascular anatomical characteristics of the lesions, patient's preference, cosmetic factors, and available expertise.

## Data Availability Statement

The original contributions presented in the study are included in the article/supplementary material, further inquiries can be directed to the corresponding author.

## Ethics Statement

The studies involving human participants were reviewed and approved by the study was approved by the Huashan Hospital Institutional Review Board (HIRB), Fudan University, Shanghai, China. The patients/participants provided their written informed consent to participate in this study.

## Author contributions

YS and PLiu: conception and design of study, collection and/or assembly of data, data analysis and interpretation, and manuscript writing. YT: conception and design of study and revision and final approval of manuscript. WZ: provision of study and revision and final approval of manuscript. PLi: manuscript writting. YL and ZL: data analysis and colleague. All authors contributed to the article and approved the submitted version.

## Funding

The Outstanding Academic Leaders Program of Shanghai Municipal Commission of Health and Family Planning (No. 21XD1400600 to WZ), National Natural Science Foundation of China (No. 82171311 to WZ), and Clinical Research Plan of SHDC (Nos. SHDC2020CR2034B to WZ and SHDC2020CR4033 to KQ).

## Conflict of interest

The authors declare that the research was conducted in the absence of any commercial or financial relationships that could be construed as a potential conflict of interest.

## Publisher's note

All claims expressed in this article are solely those of the authors and do not necessarily represent those of their affiliated organizations, or those of the publisher, the editors and the reviewers. Any product that may be evaluated in this article, or claim that may be made by its manufacturer, is not guaranteed or endorsed by the publisher.
